# Expanding the clinical spectrum of hereditary fibrosing poikiloderma with tendon contractures, myopathy and pulmonary fibrosis due to *FAM111B* mutations

**DOI:** 10.1186/s13023-015-0352-4

**Published:** 2015-10-15

**Authors:** Sandra Mercier, Sébastien Küry, Emmanuelle Salort-Campana, Armelle Magot, Uchenna Agbim, Thomas Besnard, Nathalie Bodak, Chantal Bou-Hanna, Flora Bréhéret, Perrine Brunelle, Florence Caillon, Brigitte Chabrol, Valérie Cormier-Daire, Albert David, Bruno Eymard, Laurence Faivre, Dominique Figarella-Branger, Emmanuelle Fleurence, Mythily Ganapathi, Romain Gherardi, Alice Goldenberg, Antoine Hamel, Jeanine Igual, Alan D. Irvine, Dominique Israël-Biet, Caroline Kannengiesser, Christian Laboisse, Cédric Le Caignec, Jean-Yves Mahé, Stéphanie Mallet, Stuart MacGowan, Maeve A. McAleer, Irwin McLean, Cécile Méni, Arnold Munnich, Jean-Marie Mussini, Peter L. Nagy, Jeffrey Odel, Grainne M. O’Regan, Yann Péréon, Julie Perrier, Juliette Piard, Eve Puzenat, Jacinda B. Sampson, Frances Smith, Nadem Soufir, Kurenai Tanji, Christel Thauvin, Christina Ulane, Rosemarie M. Watson, Nonhlanhla P. Khumalo, Bongani M. Mayosi, Sébastien Barbarot, Stéphane Bézieau

**Affiliations:** CHU de Nantes, Service de Génétique Médicale, Unité de Génétique Clinique, Centre de Référence Anomalies de Développement et Syndromes Malformatifs de l’interrégion Grand-Ouest, 9 quai Moncousu, 44093 Nantes CEDEX 1, France; INSERM UMR1089, Atlantic Gene Therapy Institute, University of Nantes, Nantes, France; Centre de Référence des Maladies Neuromusculaires Rares de l’Enfant et de l’Adulte Nantes-Angers, Nantes, F-44000 France; CHU Nantes, Service de Génétique Médicale, Unité de Génétique Moléculaire, 9 quai Moncousu, 44093 Nantes CEDEX 1, France; Hôpital de la Timone, Service de Neurologie, Centre de Référence des maladies Neuromusculaires et Sclérose Latérale Amyotrophique, Marseille, France; CHU de Nantes, Laboratoire d’Explorations Fonctionnelles, Nantes, F-44000 France; Department of Medicine, Columbia University Medical Center, New York, NY USA; Hôpital Necker Enfants Malades, AP-HP, Service de Dermatologie, Paris, France; Equipe d’accueil Biometadys, Université de Nantes, Nantes, France; CHU Nantes, Service de Radiologie, CHU Nantes, Nantes, F-44000 France; Service de neuropédiatrie, Hôpital Timone, Aix-Marseille Université, Marseille, France; Hôpital Necker-Enfants malades, AP-HP, U781, Fondation Imagine, Paris Descartes-Sorbonne Paris Cité, Service de Génétique, Paris, 75015 France; Centre de référence de Pathologie Neuromusculaire Paris-Est, Institut de Myologie, GHU La Pitié-Salpétrière, AP-HP, Paris, France; Equipe d’accueil EA 4271 GAD “Génétique des Anomalies du Développement”, IFR Santé STIC, Université de Bourgogne, Dijon, France; Centre de Référence Anomalies de Développement et Syndromes Malformatifs de l’interrégion Grand-Est et Centre de Génétique, Hôpital d’Enfants, CHU, Dijon, France; Laboratoire de Neuropathologie, Faculté de Médecine, CHU Timone, Marseille, France; Etablissement de Santé pour Enfants et Adolescents de la région Nantaise, Nantes, France; Department of Neurology, Columbia University Medical Center, New York, NY USA; APHP, Service d’Histologie, INSERM U841, CHU Mondor, Créteil, France; CHU de Rouen, Hôpital Charles Nicolles, Service de Génétique, Rouen, France; CHU de Nantes, Service de Chirurgie Infantile, Nantes, France; CH de Marne la Vallée, Service de Pneumologie, Jossigny, France; Department of Paediatric Dermatology, Our Lady’s Children’s Hospital Crumlin, Dublin, Ireland; National Children’s Research Centre, Our Lady’s Children’s Hospital, Dublin, Ireland; Clinical Medicine, Trinity College Dublin, Dublin, Ireland; AP-HP Hôpital Européen Georges Pompidou, Service de pneumologie, Paris, France; AP-HP, Hôpital Bichat, Service de Génétique, Paris, France; Laboratoire d’Anatomopathologie A, Faculté de Médecine, Université de Nantes, 1, rue Gaston Veil, Nantes Cedex, 44035 France; CHU Nantes, Service de Génétique Médicale, Unité de Cytogénétique, 9 quai Moncousu, 44093 Nantes CEDEX 1, France; Service de Dermatologie, Hôpital La Timone, Aix Marseille Université, Provence, France; Centre for Dermatology and Genetic Medicine, Colleges of Life Sciences and Medicine, Dentistry & Nursing, University of Dundee, Dundee, UK; Division of Computational Biology, College of Life Sciences, University of Dundee, Dundee, UK; Department of Pathology and Cell Biology, Personalized Genomic Medicine, Columbia University Medical Center, New York, NY USA; Department of Ophthalmology, Columbia University Medical Center, New York, NY USA; CHU de Besançon, Service de Génétique Médicale, Besançon, France; CHU de Besançon, Service de Dermatologie, Besançon, France; Dermatology and Genetic Medicine, University of Dundee, Dundee, UK; AP-HP, Hôpital Bichat, Service de Génétique, INSERM U976, Paris, France; Division of Neuropathology, Columbia University Medical Center, New York, NY USA; Division of Dermatology, Department of Medicine, Groote Schuur Hospital and University of Cape Town, Cape Town, South Africa; Cardiovascular Genetics Laboratory, Hatter Institute for Cardiovascular Research in Africa, Department of Medicine, Groote Schuur Hospital and University of Cape Town, Cape Town, South Africa; CHU Nantes, Clinique dermatologique, Hôtel Dieu, Place Alexis Ricordeau, 44000 Nantes, France

**Keywords:** Poikiloderma, Myopathy, Contractures, Pulmonary fibrosis, Adiposis

## Abstract

**Background:**

Hereditary Fibrosing Poikiloderma (HFP) with tendon contractures, myopathy and pulmonary fibrosis (POIKTMP [MIM 615704]) is a very recently described entity of syndromic inherited poikiloderma. Previously by using whole exome sequencing in five families, we identified the causative gene, *FAM111B* (NM_198947.3), the function of which is still unknown. Our objective in this study was to better define the specific features of POIKTMP through a larger series of patients.

**Methods:**

Clinical and molecular data of two families and eight independent sporadic cases, including six new cases, were collected.

**Results:**

Key features consist of: (i) early-onset poikiloderma, hypotrichosis and hypohidrosis; (ii) multiple contractures, in particular triceps surae muscle contractures; (iii) diffuse progressive muscular weakness; (iv) pulmonary fibrosis in adulthood and (v) other features including exocrine pancreatic insufficiency, liver impairment and growth retardation. Muscle magnetic resonance imaging was informative and showed muscle atrophy and fatty infiltration. Histological examination of skeletal muscle revealed extensive fibroadipose tissue infiltration. Microscopy of the skin showed a scleroderma-like aspect with fibrosis and alterations of the elastic network. *FAM111B* gene analysis identified five different missense variants (two recurrent mutations were found respectively in three and four independent families). All the mutations were predicted to localize in the trypsin-like cysteine/serine peptidase domain of the protein. We suggest gain-of-function or dominant-negative mutations resulting in FAM111B enzymatic activity changes.

**Conclusions:**

HFP with tendon contractures, myopathy and pulmonary fibrosis, is a multisystemic disorder due to autosomal dominant *FAM111B* mutations. Future functional studies will help in understanding the specific pathological process of this fibrosing disorder.

## Background

Poikiloderma is a dermatologic condition characterized by skin atrophy, telangiectasias, and variegated pigmentation (hypo- and hyperpigmentation). Inherited poikiloderma is a group of rare disorders including Rothmund-Thomson syndrome (RTS [MIM 268400]), the eponymous Weary form of hereditary sclerosing poikiloderma [MIM 173700], Kindler syndrome [MIM 173650], and poikiloderma with neutropenia (PN [MIM 604173]) [1–4]. A distinct autosomal dominant form of hereditary fibrosing poikiloderma (HFP) was described in a South African family of European-descent [5]. In this two-generation family, five individuals were affected by this syndrome, including poikiloderma but with the additional features of muscle contractures, and progressive pulmonary fibrosis. Clinical manifestations were poikiloderma, telangiectasia and pigmentary anomalies especially on the face and sun-exposed areas from early childhood. Muscles contractures particularly involved the ankles and feet, and together with muscle involution caused gait disturbance. Pulmonary involvement was noted during the second decade of life; progressive dyspnoea and restrictive impairment of lung function were linked to pulmonary fibrosis. We previously identified HFP with tendon contractures, myopathy, and pulmonary fibrosis (POIKTMP) as a new clinical entity and we identified the causative mutations in the *FAM111B* gene (NM_198947.3) by whole-exome sequencing [6].

Here, we extend the description of the POIKTMP phenotype through observations made in two family cases and a series of eight sporadic cases with dominant causative *FAM111B* mutations. The main features consist of (i) early-onset poikiloderma, hypotrichosis, hypohidrosis; (ii) muscle contractures with varus foot deformity; (iii) progressive proximal and distal muscle weakness and (iv) progressive pulmonary fibrosis. In total, we identified five different missense mutations that are predicted to localize in the functional domain of the FAM111B protein. Histological data showed a multisystemic adiposis and fibrosis leading to this disorder.

## Methods

### Patient recruitment

All the patients were referred by their referent physicians (geneticists, dermatologists, neurologists or pulmonologists) either on typical clinical features and/or after *FAM111B* mutation identification by whole exome sequencing. The five members of the South African family F10 and individuals F1, proband F2, F3, F4 were previously reported [5, 6]. Individuals F1, proband F2, F3 and F4 were described in the initial report as individuals F1-II2, F2-II4, F3-II1 and F4-II1, respectively [6]. In family F2, the affected son was born after the diagnosis of POIKTMP in the father. Four additional cases were identified based on typical clinical features like poikiloderma and contractures for individuals F5, F6 and F8, and on lung fibrosis for individual F9. It is worth noting that Rothmund-Thomson syndrome (RTS) was suggested for almost all the patients in the first years of life (no mutation was found in the main causative gene for RTS, *RECQL4* [MIM 603780]). Whole exome sequencing was performed in individuals F6 at the Centre for Dermatology and Genetic Medicine at the University of Dundee and F7 at the Personalized Genomic Medicine laboratory at Columbia University and identified a *FAM111B* mutation leading to the diagnosis.

### Clinical investigation and phenotype

Ethnic origin and family history information was collected. We routinely analyzed parameters such as auxology, skin examination, myopathic features and/or contractures, lung impairment or any other major medical event as shown in Table 1.

**Table 1:** Clinical and molecular data of affected individuals affected

Characteristics		Individual F1^a^	Family F2^a^		Individual F3^a^	Individual F4^a^	Individual F5	Individual F6	Individual F7	Individual F8	Individual F9	Family F10^a^			
		Origin: France	Origin: Algeria		Origin: Italy	Origin: France/Morocco	Origin: France	Origin: Ireland	Origin: Dominican Republic	Origin: France	Origin: France	South-Africa			
	Sex	M	M (proband)	M (son)	F	F	F	F	M	M	M	F (proband)	M (father)	M (brother)	M (brother)
	Age at last examination	10 yrs	32 yrs	8 months	13 yrs	9 yrs	4 yrs	5 yrs	23 yrs	8 yrs	Death: 40 yrs	26 yrs	Death: 56 yrs	Death: 30 yrs	31 yrs
	Consanguinity	No	Yes	No	No	No	No	No	No	No	No	No	No	No	No
General	Growth retardation/Hypotrophy (height; weight)	No	165 cm (−1.7 SD); 40 kg (BMI:14.7)	No	145 cm (−1.5 SD); 30 kg (BMI: 14.3)	120 cm (−1.9 SD); 15.8 kg (BMI:11)	102 cm (+0.2 SD); 14 kg (BMI:13.5)	91.3 cm (−3.9 SD); 12.3 kg (BMI:14.8)	No	No	163 cm (−2.1 SD); 53 kg (BMI:19.9)	No	No	No	No
	Delayed puberty	n/a	Yes (17 yrs)	n/a	Yes	n/a	n/a	n/a	No	n/a	No	n/a	n/a	n/a	n/a
	Normal IQ	Yes	Yes	n/a	Yes	Yes	Yes	Yes	Yes	Yes	Yes	Yes	Yes	Yes	Yes
	Psychiatric disorders	No	No	n/a	No	No	No	No	No	No	Schizophrenia	No	No	No	No
Skin	Poikiloderma (early childhood)	Yes	Yes	Yes	Yes	Yes	Yes	Yes	Yes	Yes	Yes	Yes	Yes	Yes	Yes
	- Face	Yes	Yes	Yes	Yes	Yes	Yes	Yes	Yes	Yes	Yes	Yes	Yes	Yes	Yes
	- Exposed area/photosentivity	Yes	Yes	Yes	Yes	Yes	Yes	No	Yes	Yes	Yes	Yes	Yes	Yes	Yes
	- Upper and/or lower limbs	Yes	Yes	No	Yes	Yes	Yes	Yes	Yes	Yes	No	Yes	Yes	Yes	Yes
	Bullous lesions	Yes	No	No	No	No	No	Yes	No	No	No	No	No	No	No
	Eczema-like	Yes	Yes	Yes	No	Yes	Yes	Yes	No	No	No	No	No	No	No
	Ichthyosis-like	No	No	No	No	No	Yes	No	No	No	Yes				
	Psoriasis-like	No	No	No	No	Yes	No	No	No	No	No	No	No	No	No
	Blaschko linear hypo/hyperpigmentation	No	No	No	No	No	Yes	No	Yes	No	No	No	No	No	No
	Lymphoedema of extremities	Yes	No	No	Yes	Yes	Yes	Yes	Yes	No	Yes	No	No	No	No
	Cellulitis	Yes	No	No	Yes	No	No	Yes	No	No	No	No	No	No	No
	Sclerosis of the digits	No	Yes	No	No	No	No	No	No	No	No	Yes	n/a	n/a	Yes
	Palmoplantar abnormalities	No	No	No	No	No	Palmar erythrosis and palmoplantar keratoderma	No	No	Palmar erythrosis	Palmoplantar keratoderma				
	Hypohidrosis/Heat intolerance	Yes	Yes	Yes	n/a	Yes	Yes	Yes	Yes	No	n/a	Yes	Yes	Yes	Yes
Hair	Hypotrichosis/Alopecia	Yes	Yes	Yes	Yes	Yes	Yes	Yes	Yes	Yes	Yes	Yes	n/a	n/a	Yes
	- Scalp hair	Yes	Yes	Yes	Yes	Yes	Yes	Yes	Yes	Yes	Yes	Yes	Yes	Yes	Yes
	- Eyebrows	Yes	Yes	Yes	Yes	Yes	Yes	Yes	Yes	Yes	Yes	Yes	Yes	Yes	Yes
	- Eyelashes	Yes	Yes	Yes	Yes	Yes	Yes	Yes	Yes	Yes	Yes	No	No	No	No
Nails	Dysplasia	No	No	No	Yes	No	No	Yes	Yes	No	No	Yo	No	No	No
Muscle	Muscle weakness (Age at onset)	Yes (7 yrs)	Yes (11 yrs)	No	Yes (4y)	Yes (14 months)	No	Yes (infancy)	Yes (2 yrs)	Yes (8 yrs)	No	Yes (9 yrs)	n/a	n/a	n/a
	- Proximal lower limbs	Yes	yes	No	Yes	Yes	No	Yes	Yes	Yes	No	n/a	n/a	n/a	n/a
	- Distal lower limbs	Yes	Yes	No	Yes	Yes	No	No	Yes	Yes	No	n/a	n/a	n/a	n/a
	- Proximal upper limbs	Yes	Yes	No	Yes	Yes	No	No	Yes	No	No	n/a	n/a	n/a	n/a
	- Distal upper limbs	Yes	Yes	No	Yes	Yes	No	No	Yes	No	No	n/a	n/a	n/a	n/a
	- Neck: extensors/Sternocleidomastoid (SCM)	No	Yes (SCM), trunk extensors, abdominal muscles	No	n/a	Yes	No	No	Yes	No	No	n/a	n/a	n/a	n/a
	Amyotrophy	Yes	Yes	No	Yes	Yes	No	No	No	No	No	n/a	n/a	n/a	n/a
	Abolition of lower limb tendon reflex	No (weak reflexes)	Yes	No	Yes	Yes	No	n/a	Yes	No	No	n/a	n/a	n/a	n/a
	Tendon lengthening (age)	Yes (7 yrs)	Yes (11 yrs)	No	Yes (13 yrs)	No	No	No	No	No	No	Yes (14 yrs)	No	Yes (5 yrs)	No
Joints	Lower limbs contractures(Age at onset)	Triceps surae (6 yrs)	Triceps surae (7 yrs)	No	Triceps surae	Triceps surae (2 yrs) Hamstring (7 yrs)	Triceps surae (3 yrs)	Triceps surae (4 yrs)	No	Triceps surae (7 yrs)	No	Triceps surae	n/a	n/a	No
	Triceps surae muscle/Hamstring muscle														
	Upper limb contractures	No	Biceps brachii (2–3 yrs) Carpal extensor	No	Yes	Yes	No	No	Yes (identified at age 23)	No	No	No	n/a	n/a	No
	Biceps brachii and carpal extensors														
Spine	Scoliosis	No	Yes	No	Yes	No	No	No	No	No	No	No	No	No	No
Oral sphere	Dysphagia/Velopharyngeal insufficiency	No	Yes	No	n/a	Yes	No	No	Yes	No	No	n/a	n/a	n/a	n/a
Liver	Hepatomegaly	No	No	No	Yes	No	No	No	No	No	No	No	No	No	No
Pancreas	Steatorrhea/Exocrine insufficiency	Yes	n/a	n/a	n/a	No	Yes	Yes	Yes	No	No	n/a	n/a	n/a	n/a
Eye	Cataract	No	No	No	Yes	No	No	No	No	No	No	No	No	No	No
	Other	No	No	No	No	No	No	No	Shallow orbits with mild restriction of medial rectus action OU; right macular pigmentary changes	No	Corneal thickness	No	No	No	No
Blood test	SCK (UI/l) (maximum)	N	460	n/a	500	340	370	N	372	n/a	N	n/a	n/a	n/a	n/a
	Blood count abnormalities (maximum)	Eosinophilia 1.5 × 10e9/L	N	n/a	N	Eosinophilia 0.8 × 10e9/L	N	Eosinophilia 0.9 x 10e9/L	PLT count: 78 x 10e9/L, MCV: 98.4 fL	N	N	n/a	n/a	n/a	n/a
	Liver function	n/a	N	n/a	Cholestasis	n/a	SGOT: 63 IU/L (<53); SPGT: 56 IU/L (<36); ALP: 308 IU/L (<335); GGT: 53 IU/L (<26)	SGOT:210 IU/L (<40); SGPT: 151 IU/L (<35); ALP: 772 IU/L (<315); Bili: 33 mmol/l (<14)	SGOT: 100 IU/L (<38); SGPT: 132 IU/L (<41); ALP:129 IU/L (<129); GGT:106 IU/L (<58)	n/a	n/a	n/a	n/a	n/a	n/a
Muscle exploration	EMG: myogenic	Yes	n/a	n/a	Yes	Yes	n/a	n/a	N	n/a	n/a	n/a	n/a	n/a	n/a
	MRI/CT-scan	Adiposis (MRI)	Adiposis (MRI)	n/a	n/a	n/a	n/a	n/a	Atrophy of paraspinal and rectus abdominis muscles (CT scan)	n/a	Adiposis (MRI)	n/a	n/a	n/a	n/a
Lung exploration	PFT (pulmonary Function Test): Restrictive syndrome	Yes (asthma)	Yes	n/a	n/a	Yes	n/a	Yes	Yes	Yes (poor participation)	Yes	Yes	Yes	Yes	No
	- FVC (% of predicted)	1.61 L (83 %)	1.82 L (42 %)	n/a	n/a	38 %	n/a	53 %	64 %	1.33 L (86 %)	1.47 L (36 %)	78 %	n/a	34 %	91 %
	- FEV1 (% predicted)	1.25 L/min (73 %)	2.02 (44 %)	n/a	n/a	n/a	n/a	56 %	n/a	1.32 L/min (101 %)	1.15 L/min (34 %)	n/a	n/a	37 %	n/a
	- FEV1/FCV (%)	78 %	111 %	n/a	n/a	n/a	n/a	N	n/a	99 %	78 %	86 %	n/a	90 %	76 %
	- DLCO	n/a	41 %	n/a	n/a	51 %	n/a	n/a	n/a	0.87 (64 %)	Not feasible	67 %	n/a	34 %	88 %
	PET/CT-scan	n/a	No pulmonary fibrosis (CT scans)	n/a	n/a	No fibrosis	n/a	No fibrosis	No pulmonary fibrosis; presence of a nodule	n/a	Pulmonary fibrosis, slightly hypermetabolic lesions	n/a	n/a	n/a	n/a
Pathology	Peripheral muscle	Adiposis	Dystrophy, fibrosis, adiposis	n/a	Dystrophy, fibrosis, adiposis	Fibrosis, adiposis	n/a	n/a	Fibroadipose replacement, endomysial fibrosis, atrophic and hypertrophic fibers, central nuclei	n/a	n/a	n/a	n/a	Fatty infiltration	n/a
	Skin	Sclerodermiform aspect, Elastin anomalies	n/a	n/a	n/a	RTS-like	n/a	Hyperkeratosis, parakeratosis, hypergranulosis, acanthosis, spongiosis. Numerous apoptotic keratinocytes.	n/a	n/a	n/a	n/a	n/a	Sclerodermiform aspect, fibrosis, elastic tissue degeneration	n/a
	Visceral organs	n/a	n/a	n/a	n/a	n/a	n/a	n/a	n/a	n/a	n/a	n/a	Pulmonary fibrosis	Pulmonary, esophageal and mediastinal lymph node fibrosis, pancreas fatty infiltration	n/a
	Vasculature	n/a	n/a	n/a	n/a	n/a	n/a	n/a	n/a	n/a	n/a	n/a	n/a	Elastic degeneration, medial calcification	n/a
Gene analysis	*RECQL4*	No mutation	No mutation	n/a	No mutation	No mutation	No mutation	No mutation	No mutation	No mutation	No mutation	n/a	No mutation	No mutation	n/a
	Other genes	No	*CAPN3*, *LMNA*, *CAV3 (no mutation)*	*No*	No	*SMN1 (no mutation)*	No	No	*CLCN1- c.2509-3C > T (intronic between exons 22 and 23); c.2926 C > T (p.976 R > X, nonsense mutation)*	No	No	No	No	No	No
	*FAM111B*	c.1879A > G (p.Arg627Gly)	c.1879A > G (p.Arg627Gly)	c.1879A > G (p.Arg627Gly)	c.1879A > G (p.Arg627Gly)	c.1883G > A (p.Ser628Asn)	c.1883G > A (p.Ser628Asn)	c.1883G > A (p.Ser628Asn)	c.1883G > A (p.Ser628Asn)	c.1874C > A (p.Thr625Asn)	c.1289A > C (p.Gln430Pro)	c.1861 T > G (p.Tyr621Asp)	n/a	c.1861 T > G (p.Tyr621Asp)	c.1861 T > G (p.Tyr621Asp)
	Inheritance	*De novo*	*De novo*	Paternal inheritance	n/a	No maternal inheritance	*De novo*	*De novo*	*De novo*	*de novo*	n/a	Paternal inheritance	n/a	Paternal inheritance	Paternal inheritance

^a^Individuals F1, proband F2, F3 and F4 were described in the initial report as individuals F1-II2, F2-II4, F3-II1 and F4-II1, respectively [6]

The following abbreviations are used: *ALP* alkaline phosphatase, *Bili* bilirubin, *BMI* body mass index, *DLCO* diffusing capacity of the lung for carbon monoxide, *EMG* electromyography, *FEV1* forced expiratory volume, *FVC* forced vital capacity, *GGT* gamma-glutamyl transpeptidase, *MCV* mean corpuscular volume, *N* normal, *n/a* not available, *MRI* magnetic resonance imaging, *PET/CT-scan* positron emission tomography/computed tomography scan, *PFT* pulmonary function test, *PLT* platelet, *RTS* Rothmund-Thomson syndrome, *SCK* serum creatine kinase, *SCM* sternocleidomastoid muscle, *SGOT* serum glutamate oxaloacetic transaminase, *SGPT* serum glutamate pyruvate transaminase

### Consent

This study was approved by the institutional review board of the Hospital of Nantes and other contributing institutions. Written informed consent was obtained from each adult participant and the parents of the participating children.

### *FAM111B* gene identification and sequencing

As described in Mercier et al., 2013, a whole exome-sequencing strategy applied to two independent families of Caucasian descent, a simplex French one, F1, and a multiplex South African one, F10, highlighted the involvement of *FAM111B* mutations in POIKTMP [6]. More recently, we found *de novo* mutations in five additional independent cases: three were revealed by either high-throughput (with a minimal 100× read depth), and/or Sanger sequencing targeting *FAM111B*, and the two others were identified by whole-exome sequencing. In each family, parents’ samples were analysed for screening of the familial mutation when available, which enabled confirmation of the *de novo* nature of the variant encountered (Table 1). Besides, the absence of the *FAM111B* variants observed in POIKTMP patients was checked by Sanger sequencing in 388 healthy controls from different ethnic origins (including 96 Algerians, 127 Moroccans and 165 South Africans).

## Results

### Demographic data

Fifteen patients belonging to 10 independent families were diagnosed with POIKTMP (Table 1). A mutation in the *FAM111B* gene was identified in all the patients tested. Six were females and nine were males. They were of French, Algerian, Irish, Italian, Moroccan, Dominican Republic and South-African origins. The median age at last follow-up was 20.5 years (min = 8 months; max = 56 years).

### Poikiloderma and ectodermal abnormalities

**Fig. 1:**
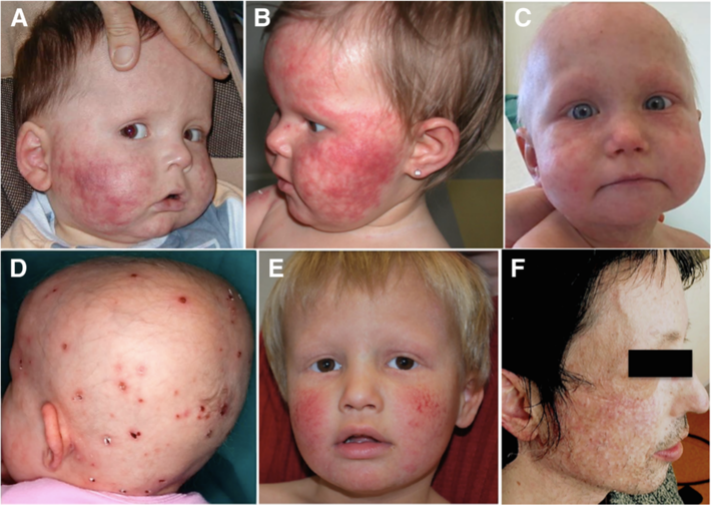
Facial and scalp skin lesions. Poikiloderma and alopecia of the scalp, eyebrows, and eyelashes in individuals F1, F4, F5, F6, F8 and F9 (**a**-**f**)

**Fig. 2:**
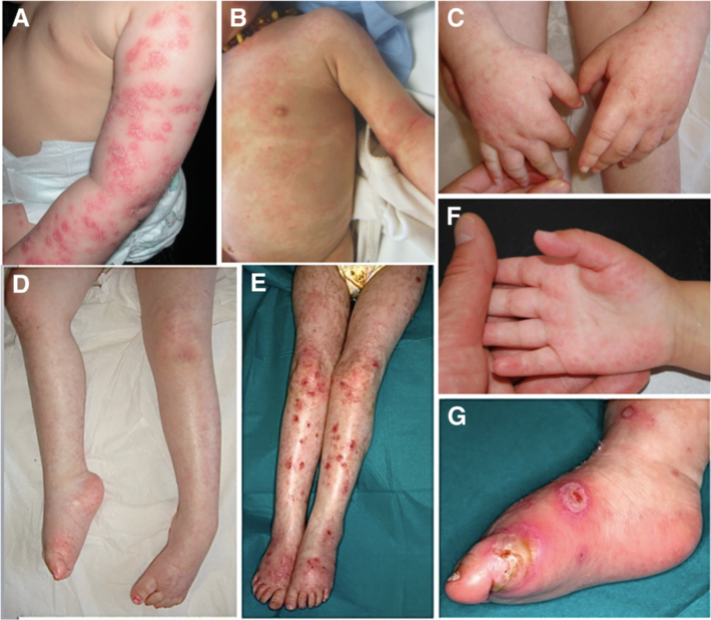
Skin lesions of upper and lower limbs. Eczema-like and psoriasis-like dermatosis of the upper limbs in individual F4 (**a**); hyperpigmentated regions in individual F5 (**b**); chronic lymphoedema of lower limbs and hands in individuals F4 (**c**, **d**), F6 (**e**), F8 (**f**); diffuse skin lesions of lower limbs and cellulitis in individual F6 (**e**, **g**)

Skin abnormalities were the earliest findings in all patients. Poikiloderma appeared during early infancy, typically in the first six months of age. It was mainly localized to the face (Fig. 1). Transient exacerbations of facial erythema were seen following sun exposure. This erythema was complicated by bullous lesions in individual F1. Hyperpigmented and hypopigmented lesions are a constituent part of poikiloderma but individual F5 also had Blaschko linear hyperpigmentation (Fig. 2b).

Sparse scalp hair, sparse or absent eyelashes and/or eyebrows was found in all patients with variable severity. Hair dysplasia and leukoplakia were not observed (Fig. 2). Three patients had nail dysplasia. Hypohidrosis with heat intolerance was observed in most of the patients (11/12).

In addition, seven patients had lymphoedema of lower and/or upper extremities that was complicated by cellulitis in three of them (Fig. 2g). Chronic erythematous and scaly skin lesions described by clinicians as eczema-like, ichthyosis-like or psoriasis-like lesions were often observed on the limbs. Some patients had palmoplantar erythrosis, mild palmoplantar keratoderma or sclerosis of the digits. Of note, skin lesions in particular facial poikiloderma improved with time.

**Fig. 3:**
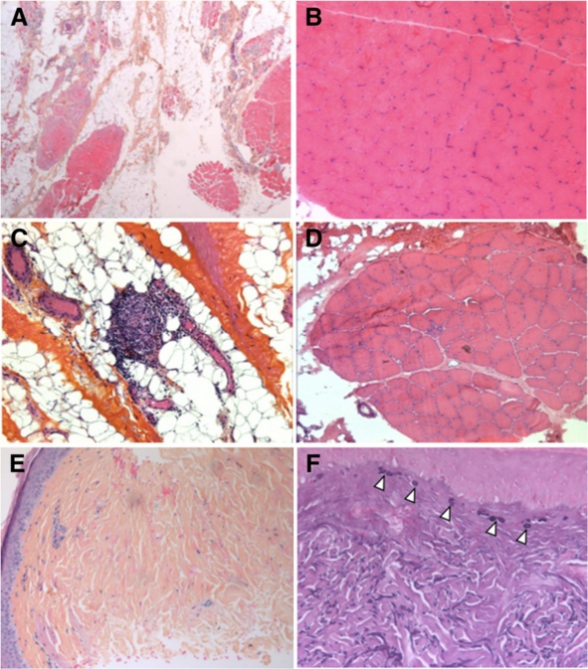
Muscle and skin microscopy. **a**-**d** Fatty tissue, fragmented muscle fascicles next to normal fascicles in individuals F1 (**a**-**b**) or nonspecific myopathic changes with variation in fiber size in proband F2 (**c**-**d**) (Hematoxylin and eosin staining [H&E]; ×20 (**a**) and ×100 magnification (**b**-**d**)). **e**-**f** Epidermal atrophy, scleroderma-like features with a diffuse mild collagen sclerosis (**e**) (individual F1; H&E; ×20 magnification); elastic dystrophy with formation of elastic globes (*arrowheads*) in the papillary dermis (**f**) (individual F1; Weigert staining; ×150 magnification)

Microscopic examination of the biopsied skin performed in four patients revealed a very characteristic pattern of epidermal atrophy with scleroderma-like features and conspicuous alterations of the elastic network in the superficial and deep dermis. Enlarged and fragmented elastic fibres were noted and the formation of elastic globes in the papillary dermis was associated with a diffuse slight collagen sclerosis (Fig. 3). Lesions could easily be misdiagnosed as RTS lesions, as it was the case for individual F4. In individual F6, hyperkeratosis, parakeratosis, hypergranulosis were observed as well as acanthosis and spongiosis with numerous apoptotic keratinocytes.

### Muscle contractures and myopathy

After cutaneous features, muscle contractures represent the second suggestive finding of POIKTMP. These can be seen as early as 2 years of age in some patients. The most commonly affected muscle was the triceps surae, leading to a shortening of Achilles tendons. Five patients underwent surgery for Achilles tendon lengthening at median age of 10 years (min = 5; max = 14). For example, individual F1 had very severe varus deformities of both feet. His gait impairment appeared at the age of 6 years and outdoor wheelchair use was required by the age of 7 years. Following tendon lengthening surgery, he was able to walk again. In four patients, contractures of upper limbs (biceps brachii and carpal extensors) were also noted. Thoracolumbar scoliosis was noticed in two patients (proband F2 and F3).

**Fig. 4:**
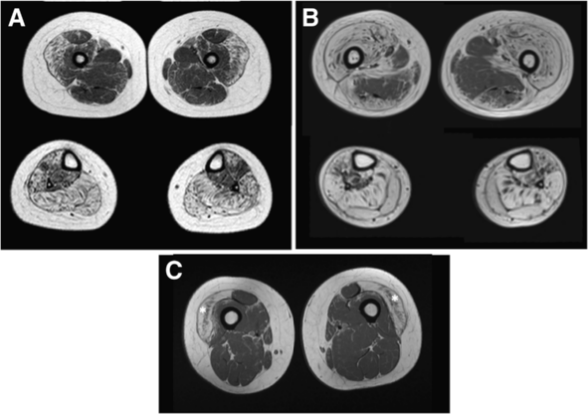
Muscle MRI (coronal images: thighs (*up*); calves (*down*); T1-weighted sequence). Diffuse bright appearance of the anterior compartment of the thighs, particularly in the vastus lateralis muscles, and the posterior compartment of the calves in individual F1 at 7 years of age (**a**); more severe stage with a relative sparing of the posterior compartment of the thighs in proband F2 at 30 years (**b**). Specific involvement of the vastus lateralis muscles (*asterisks*) with sparing of other thigh muscles in individual F9 (**c**)

Muscle atrophy was observed in four patients (individuals F1, F3, F4 and proband F2) and in some cases, was diffuse and severe. The South African proband had atrophic thenar and hypothernar eminences. Similarly, individual F1 did not have the ability to oppose the thumb on both hands. The majority of patients (8/11) developed a progressive weakness of both proximal and distal muscles of all four limbs, although the first symptoms observed in lower limbs were rather proximal. The median age of onset of muscle involvement was 5.9 years (min = 1; max = 11). Clinical variability of muscle weakness was high. The most severe case, individual F4, lost ambulation at the age of 3 years old. At the age of 9 years she could no longer transfer from bed to wheelchair. Her muscle strength (MRC score) was graded globally between 2 and 3. The other individuals are still ambulatory. Of the adult patients, proband F2 was able to walk only a few steps before stopping and was unable to climb stairs at 31 years of age. He presented with a pronounced axial muscle impairment especially in the abdominal belt, trunk and neck extensors with dropped head. In contrast, muscle strength testing did not show any weakness in the 40-year-old individual F9. However, muscle involvement was confirmed in this individual by muscle MRI, which showed a selective involvement of the vastus lateralis muscles (Fig. 4). Muscle MRI was performed in two other patients (individuals F1 and proband F2), respectively at age 7 and 30. The older individual F2 had more severe muscle impairment. The MRI revealed a severe diffuse fatty infiltration of legs with a relative sparing of tibialis posterior and a severe fatty infiltration of the anterior compartment of thighs with a relative sparing of posterior compartment. Abdominal CT scan performed in individual F7 revealed atrophy of the paraspinal and rectus abdominis muscles.

Serum creatine kinase was either normal (in 3/8 patients) or slightly increased (5/8 patients; max = 500 IU/L). When performed, electromyography showed a normal or myopathic pattern.

Muscle biopsy performed in six patients revealed the same histopathologic pattern (Fig. 3). There was extensive fatty infiltration and residual muscle tissue was composed of fragmented muscle fascicles with either normal fibers or atrophic fibers with central nuclei. No neuropathic features (i.e. normal ATPase pattern) or mitochondrial network abnormalities were found on histochemistry or immunolabelling. Western blot analysis in proband F2 showed a secondary reduction of calpain.

### Pulmonary impairment

All patients for whom pulmonary data were available had abnormal lung function with a restrictive pulmonary pattern. Non-invasive ventilation was considered for proband F2 who had a severe restrictive pulmonary pattern. Individual F4 had recurrent bronchitis. Progressive interstitial pulmonary fibrosis was not observed in children, and found in only half of the adults (3/6): individual F9 and two men from the original South African family. Pulmonary complications were life-threatening in some individuals, as seen in these three individuals who died at 30, 40 and 56 years of age. Two of them died only three or four years after the first respiratory symptoms such as progressive breathlessness and dry cough.

### Other systemic features

#### Growth retardation and delayed puberty

Growth retardation and/or hypotrophy were observed in six individuals with delayed puberty in two individuals. Enteral feeding was required in individual F4 due to low weight, which remained 15 kg (<−3 SD) at 6 years of age. In this individual, IGF1 level was low at 58 μg/L (95–240). No specific testing was performed in the other patients to investigate this feature.

#### Liver involvement

Liver impairment was reported in four patients. Individuals F5, F6 and F7 initially presented with mildly elevated transaminases, alkaline phosphatises, gamma-glutamyl transferase, and/or bilirubin, which fluctuated between normal and abnormal for F7. Furthermore, individual F3 had hepatomegaly and cholestasis, which was treated with ursodesoxycholic acid.

#### Pancreatic exocrine insufficiency

Pancreatic exocrine insufficiency was diagnosed in four individuals (F1, F5, F6 and F7). Symptoms included fatty stools and diarrhea, which normalized with pancreatic enzyme supplementation. A CT scan of abdomen showed severe pancreatic atrophy in individual F7. It is worth noting that post-mortem examination of one individual of the South African family F10 showed extensive fatty infiltration of the pancreas [5].

#### Ophthalmologic findings

Some ophthalmologic abnormalities were observed in three individuals viz: cataracts in individual F3, shallow orbits with mild restriction of medial rectus action OU and right macular pigmentary changes in individual F7 and corneal thickness in individual F9. No other ophthalmologic findings were associated.

#### Neurodevelopment

Cognitive development and function were totally normal in all patients. Of note, one individual (F9) had schizophrenia. No other psychiatric disorders were reported.

#### Haematological abnormalities

Eosinophilia was observed in three patients (individuals F1, F4 and F6). Individual F7 had a mild thrombocytopenia and a slightly increased mean corpuscular volume.

### Genetics

To determine the molecular basis of POIKTMP, a whole-exome sequencing strategy was first applied to the French family F1 and the South African F10 as described in Mercier et al., 2013. *FAM111B* (NM_198947.3) appeared as the only candidate gene in common between the two families: c.1879A > G (p.Arg627Gly) was the unique *de novo* variant found in the individual F1 and c.1861 T > G (p.Tyr621Asp) was observed in the affected individuals of family F10.

**Fig. 5:**
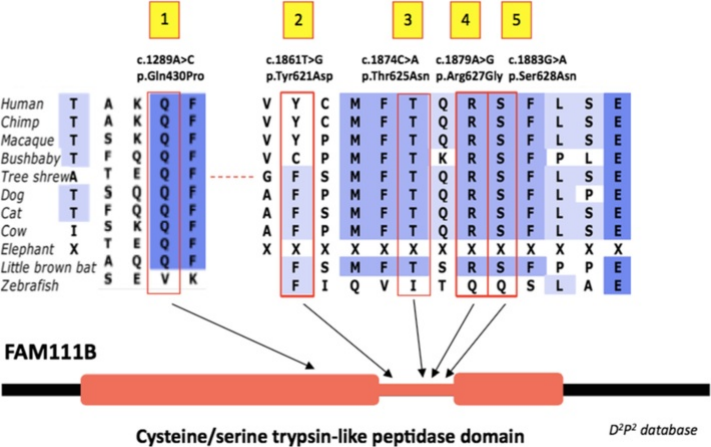
Missense variants identified in *FAM111B*. Conserved amino acid sequences among mammals and cluster within a putative cysteine/serine trypsin-like peptidase domain of FAM111B. Variant #1 [c.1289A > C (p.Gln430Pro)] identified in individual F9; variant #2 [c.1861 T > G (p.Tyr621Asp)] in the South-African family F10; variant #3 [c.1874C > A (p.Thr625Asn)] in individual F8; variant #4 [c.1879A > G (p.Arg627Gly)] in individuals F1, F3 and family F2; variant #5 [c.1883G > A (p.Ser628Asn) in individuals F4, F5, F6 and F7

We identified a causative mutation in each family of the series. The variants consist of five different missense mutations that are predicted to localize in the trypsin-like cysteine/serine peptidase domain of the protein (Fig. 5) and are absent from all public genetic variant databases tested (dbSNP142 (http://www.ncbi.nlm.nih.gov/snp/); Exome Variant Server, NHLBI GO Exome Sequencing Project (ESP), Seattle, WA (URL: http://evs.gs.washington.edu/EVS/) [accessed on October 2015]; Exome Aggregation Consortium (ExAC), Cambridge, MA (URL: http://exac.broadinstitute.org) [accessed on October 2015]; Genome of the Netherlands [7]) and from 388 healthy controls of different ethnic origin. Four variants are within seven consecutive codons and encode amino acids located in the loop of the predicted functional domain of FAM111B: c.1861 T > G (p.Tyr621Asp), c.1874C > A (p.Thr625Asn), c.1879A > G (p.Arg627Gly), and c.1883G > A (p.Ser628Asn). The last variant identified in individual F9 is located upstream of the loop, but still in the trypsin-like cysteine/serine peptidase domain (c.1289A > C (p.Gln430Pro)). Mutations reported in POIKTMP are available in the LOVD variant database dedicated to *FAM111B* (www.LOVD.nl/FAM111B).

## Discussion

We report a series of ten families of HFP with muscle contractures, myopathy, and pulmonary fibrosis due to dominant mutations in the *FAM111B* gene. Here we have added six new cases to the previously reported cases and confirm that POIKTMP is a multisystemic disorder involving the processes of fibrosis and adiposis [5, 6].

In our series, all patients had poikiloderma in early infancy, which is a key feature in diagnosing this disorder. The phenotypes presented here are distinct from other types of hereditary poikiloderma, such as RTS, hereditary sclerosing poikiloderma of Weary, Kindler syndrome or poikiloderma with neutropenia [1–4, 8–10]. In our cases, skin lesions improved with time whereas extracutaneous manifestations became more prominent. RTS is the main differential diagnosis for POIKTMP and most patients were initially misdiagnosed as RTS in childhood. POIKTMP and RTS share the following features: early-onset poikiloderma, ectodermal dysplasia features (hypotrichosis, hypohidrosis and nail dysplasia), palmoplantar keratoderma, growth delay, cataracts and haematological abnormalities [1].

Strikingly, myopathy appears to be specific to POIKTMP. Some patients presented with early prominent joint contractures, mainly in the triceps surae muscles. The severity of these contractures could impair the gait and Achilles tendon lengthening was performed successfully in affected patients. The patients with muscle involvement developed weakness in both distal and proximal leg muscles and in some of them muscle weakness extended to the upper limbs. Axial muscle involvement was also present, especially in the trunk extensors, neck flexors, abdominal belt and respiratory muscles. Muscle MRI is an easy and non-invasive procedure that can help identifying muscle involvement. Taking into account the small number of patients, MRI images revealed an early and selective involvement of the vastus lateralis muscle while posterior compartment of thighs were relatively spared. In the legs, a prominent fatty infiltration was observed in the posterior compartment while the tibialis posterior was spared. Proximodistal and axial muscle weakness was prominent in the lower legs with prominent joint contractures. Selective muscle involvement in muscle imaging in the context of poikiloderma should prompt diagnostic consideration of POIKTMP. Pulmonary fibrosis, liver and pancreatic impairment have also only been reported in POIKTMP. Some other findings such as congenital malformations (skeletal anomalies, visceral malformations) and cancer predisposition seem to be specific to RTS and are not described in POIKTMP to date.

As shown in our series, there is variability in the clinical features of POIKTMP. Some patients exhibit only cutaneous abnormalities with or without muscle involvement. We cannot predict the course of the disease and there may be long-term complications in the six patients who are younger than 13 years of age. Pulmonary fibrosis seems to affect adults only, even if a restrictive lung pattern is detected in childhood. The clinical course of pulmonary fibrosis may be rapidly progressive, as observed in individual F9 and the proband’s brother in family F10. Some patients only had restrictive lung function due to respiratory muscle involvement in the absence of pulmonary fibrosis. Regular lung function testing with monitoring of vital capacity and DLCO is recommended in the follow-up of these patients. Exocrine pancreatic insufficiency is also part of the disease. It was observed in four individuals (F1, F5, F6 and F7) and a pancreatic fatty infiltration was identified in the South African man on post-mortem examination at 30 years of age.

In the literature, two articles have described pancreatic insufficiency with fatty pancreatic degeneration in RTS-like individuals without any mutation in the *RECQL4* gene [11, 12]. Meier et al. reported in 2012 a woman with RTS who died at the age of 42 years, after multiple organ failure, including chronic end-stage renal disease, exocrine pancreatic insufficiency, lung fibrosis and lethal respiratory insufficiency due to progressive systemic muscular atrophy [11]. Of note, an abdominal CT scan showed fatty pancreatic degeneration responsible for the exocrine pancreatic insufficiency. Otsu et al. described in 2008 a 20-year-old male with exocrine pancreatic hypofunction caused by atrophy and fatty replacement of the pancreas [12]. In both cases, no mutation in *RECQL4* gene was found [12]. We suggest that these two cases were probably due to mutations in *FAM111B*. Another RTS-like case is highly suggestive of POIKTMP in a female affected with epilepsy [13]. We found neurological features only in one individual (F9) who presented with schizophrenia. It is unclear if this is an incidental association or if there is a causal link with POIKTMP. The frequency of schizophrenia is as high as 1 % in the general population and thus no conclusion can be made based on this single observation [14]. The phenotypes of POIKTMP and RTS are overlapping and the frequency of POIKTMP is probably underestimated today. We recommend *FAM111B* screening in the evaluation of RTS or more widely of early-onset poikiloderma when no mutation is found in the *RECQL4* gene (Küry et al., accepted in EJHG) [15].

As previously reported, the other major features of POIKTMP are the fatty muscle infiltration observed in muscle and skin biopsies (Figs. 3 and 4). Histological examination of skeletal muscle showed a partial loss of muscle tissue associated with an extensive fibrofatty tissue infiltration. There was no patent indication of denervation, necrosis, or inflammation (except for one isolated focus of inflammatory cells in individual F2). In the skin biopsy, the pathology studies revealed collagen sclerosis, elastic degeneration, and the absence of fatty infiltration, in contrast to the observation made in muscle tissue. In addition, the post-mortem study of one affected member of the South African family revealed a diffuse fatty infiltration and fibrosis of organs such as the lungs, oesophagus and pancreas. Similarly, the cholestasis and hepatomegaly observed in individual F3 could likely be related to such histological lesions, even if we do not have the confirmation of this assumption. In individuals F5 and F7, SGOT and SGPT may be elevated in parallel with creatine kinase as these enzymes are also found in muscle. However, the gamma-glutamyl transferase was also elevated and is specific to the hepatobiliary system. Liver blood tests were also abnormal in individual F6 with normal creatine kinase. These data are rather in favour of a liver impairment as shown in individual F3. In sum, the clinical, MRI and histological findings support a pathological process of multisystemic fibrosis and adiposis underlying this disorder.

*FAM111B* mutations were found in all the families. In individual F7, two additional variants were identified by whole exome sequencing in *CLCN1*, the gene implicated in Myotonia Congenita [MIM 255700]. We do not know if the combination of these variants could be pathogenic but the patient had no myotonia, nor myotonic discharges at EMG. Within the “family with sequence similarity 111” gene family, there are two members: *FAM111A* and *FAM111B* (NM_198947.3). In the literature, one article suggests the *FAM111A*-*FAM111B* locus to be involved in prostate cancer susceptibility [16]. Bioinformatic tools predict the FAM111B protein to contain a trypsin-like cysteine/serine peptidase enzymatic domain. This domain is 45 % homologous to the one predicted in FAM111A [MIM 615292], in which causative mutations have been recently reported to cause the Kenny-Caffey syndrome (KCS [MIM 127000]) and osteocraniostenosis (OCS [MIM 602361]), two clinical entities phenotypically distinct from POIKTMP [17]. It is worth noting that the *FAM111B* mutations are also located in the functional domain of the protein and consist of missense dominant mutations as well. This suggests either a gain-of-function or a dominant-negative effect.

FAM111B mRNA expression has been detected in many tissues, including keratinocytes, skeletal myocytes, adipose tissue and lung. We performed immunoblot analyses of tissue samples in individual F1 and detected FAM111B in skeletal striated muscle, but not in fibroblasts or in the control [6]. Functional studies are ongoing to determine the still-unknown function of FAM111B and the pathophysiological mechanisms underlying this disorder. This will hopefully lead to the identification of the pathway involved in POIKTMP pathogenesis. Advances in this area will be crucial to understand POIKTMP pathology with the aim of finding a treatment in the future. It could also be beneficial for other disorders like scleroderma or myopathies in which fibrosis and adiposis are also.

Some genotype-phenotype correlations are apparent from our observations. The most upstream mutation (codon 430) was located outside the loop of the functional domain and was identified in individual F9 who had asymptomatic muscle involvement, but severe pulmonary fibrosis in adulthood. These clinical features are very similar to the description of the South African adult patients whose mutation (codon 621) is located in the loop also upstream to the other *FAM111B* mutations. The mutations in codons 625, 627 and 628 were found in patients with an earlier onset of the disease and a more severe phenotype in terms of cutaneous, muscle and/or visceral findings. Long-term follow-up of these patients will be helpful in generating a more complete picture of this syndrome. Further case reports and case series will be needed to confirm these preliminary genotype-phenotype correlations. We postulate that mutations in other regions of FAM111B might lead to phenotypes different from POIKTMP, as observed in many other disorders [18, 19].

## Conclusions

In conclusion, we describe in the largest series to date the specific features of POIKTMP: early-onset poikiloderma, ectodermal dysplasia features, muscle contractures, myopathy, pulmonary fibrosis, as well as growth retardation, liver impairment, exocrine pancreatic insufficiency, cataracts and haematological abnormalities. An obscure process leading to adiposis and fibrosis is responsible for this multisystemic disorder due to *FAM111B* dominant mutations. Functional studies are ongoing to understand the pathological process in POIKTMP, which could also be beneficial for the understanding of other fibrotic disorders.
